# Physical Activity Pattern and Personal-Social Factors of Mothers During Pregnancy And Infant Birth Weight Based On MET Scale: A Case-Control Study

**DOI:** 10.5812/ircmj.11665

**Published:** 2013-07-05

**Authors:** Zohreh Mahmoodi, Masoud Karimlou, Homeira Sajjadi, Masoumeh Dejman, Meroe Vameghi, Mahrokh Dolatian, Monir Baradarn Eftekhari

**Affiliations:** 1 Social Determinant of Health Research Center, University of Social Welfare and Rehabilitation Sciences, Tehran, IR Iran; 2 Department of Midwifery, Shahid Beheshti University of Medical Sciences, Tehran, IR Iran; 3 Researche and Technology, Ministry of Health and Medical Education, Tehran, IR Iran

**Keywords:** Pregnancy, Motor Activity, Infant, Low Birth Weight, Effective Social Determinants of Health

## Abstract

**Background:**

Low birth weight is one of the most important public health issues in developing and developed countries and identifying its etiology is important for prevention.

**Objectives:**

Considering the unknown impact of physical activity on low birth weight, this research was conducted to investigate the relationship between physical activity and low birth weight.

**Patients and Methods:**

This research was conducted using a case-control design. The control group was made of 500 women with normal birth weight infants and the case group included 250 women with low birth weight infants from the selected hospitals in city of Tehran. The information was gathered using a researcher-made questionnaire which was prepared for determining the relationship between mothers’ lifestyle during pregnancy and infants' low birth weight using social determinants of health approach. In this questionnaire, scope of physical activity was investigated in three groups of athletic activities, activities at home and leisure activities. Activity intensity was determined using MET scale and the data were analyzed in SPSS software using independent t-test, Chi-square and logistic regression.

**Results:**

In the present research, based on the results of multiple logistic regression test, an increase in the time spent on sport activities (OR = 2.20) and home activities (OR =1.44) (P = 0.003) was accompanied by increased chance of giving birth to low birth weight infants; in contrast, one hour increase of leisure activities decreased the probability of low birth weight infants by 0.32 (P = 0.008).

**Conclusions:**

An increase in the time spent on sport and home activities, even after considering other influential factors, was related to low birth weight.

## 1. Background

Low birth weight is one of the most important public health issues in both developing and developed countries. It is one of the determining factors of survival and future physical and mental development of every infant. In fact, it is the most important, simple, common, sensitive and reliable health index used for evaluating health status of infants, hence the status of individuals and society in each country(1, 2). Low birth weight (LBW) has been defined by WHO as birth weight of less than 2500 g(3). This parameter is also divided into two groups based on gestational age; if the infant is born between 37th and 42nd weeks of pregnancy, it is low weight and mature (LBW), but if it is born before 37th week of pregnancy, it will be low weight and premature (PLBW). Low birth weight infants are more exposed to risks such as cerebral palsy, mental retardation, neurological disabilities, respiratory disorders, sudden death syndrome and complications of hospitalization in intensive care unit as compared with normal birth weight infants(3-8). In addition to mental-physical problems, cost of treating and care of these infants is six times as much as that for other infants (9).

Different factors may be related to low birth weight. According to some researchers, it may be caused by several factors including mothers' low weight at the beginning of pregnancy, mothers' short stature and illnesses like hypertension or genital infections. Many health indexes such as children’s death have improved in the recent two decades due to many efforts for controlling these biological elements, but the prevalence of low birth weight has still remained the same or even has increased in some countries(2). This point necessitates attention to the role of other factors, especially social factors and the strategies related to improving health status. In some studies in Iran, mortality determinants of infants have been identified as economic status of families, mothers’ literacy level, living in villages and high-risk birth intervals, respectively. Nevertheless, provincial comparison of infant mortality has demonstrated higher mortality rates in infants of poorer provinces, which have more prevalence of illiteracy and are mostly villager (10). These elements affect health of mothers and infants in different ways. Among these effective social determinants of health, lifestyle and its dimensions are more important in emergence and prevention of most problems of mothers during pregnancy(11). Of different aspects of lifestyle, physical activity is considered in this study(12).

According to the WHO, physical activity is defined as any bodily movement of skeletal muscles which leads to energy consumption(13). Some studies have confirmed adverse effects of mother’s high physical activity during both work and leisure on undesirable pregnancy outcomes like low birth weight. Also, some other studies have found a relationship between insufficient activity in leisure and these consequences(2). Some authors like Fox et al. (2007) did not find a significant relationship between occupational physical activity and premature delivery and some others like Saurel (2004) observed this relationship. In Iran, a study was conducted by Mehran et al. in which no relationship was reported between premature delivery and physical activity(13-18). Most women believe that physiological limitations caused by pregnancy deprive them of social programs like exercises and some others believe that resting and avoiding sport activities or active lifestyle during pregnancy are the most important factors(19). Therefore, considering these differences and unknown effect of physical activity on low birth weight, and that no study has been conducted on physical activity as a determinant besides other related factors, this study was conducted with this approach.

## 2. Objectives

The present work is a part of a large study titled "designing measuring tool and relationship pattern of mothers’ lifestyle during pregnancy and low birth weight", conducted in two steps.

## 3. Materials and Methods

This case-control study was conducted in Tehran (IRAN) in 2012. The data collection tool of the study was a researcher-made questionnaire, designed for measuring lifestyle with the approach of social determinants of health. For psychometrics of the questionnaire, face and content validities (both qualitative and quantitative methods), criterion validity by criterion tool(11) and construct validity (exploratory factor analysis) were used. The questionnaire contained 132 items in 10 sections: three sections covered general characteristics, pregnancy history and lab test results recorded in the files and seven other sections including physical activity, occupation, nutrition, stress control, self-care, social relations and inappropriate health behaviors. Cronbach's alpha coefficient also confirmed high internal consistency of the questionnaire (0.76) (20). In this study, the results related to physical activity were studied by 11 items and MET scale was presented in three parts including physical activities at home (cooking, housekeeping, babysitting and shopping), leisure activities (studying, listening to music, resting and attending religious ceremonies) and sport activities (walking, swimming, dancing and aerobics). This index measured the amount of consumed energy at the time of activity relative to that consumed at resting time.

For this case-control study, first, city of Tehran was divided to five geographical zones of north, south, east, west and center. Then, out of the hospitals in every zone that had maternity ward, some clusters including one or two governmental or social security hospitals were selected according to their delivery rates. To determine the required sample size, after reviewing the literature, considering 10% prevalence for low birth weight, and calculating the research variables, the number of items in the measurement tool and key concepts, were determined as 3 to 10 samples for each variable (21). Accordingly, 250 infants were allocated in the case group (infants with weight of less than 2500 g) and 500 infants were placed in the control group (infants weighing more than 2500 g). The inclusion criteria included.

### 3.1. Mothers

15-45-year-old Iranians at gestational age of 37-42 weeks based on the first day of their last menstruation period (LMP) or Sonography, who went to the selected hospitals for delivery.

Lack of problems like multiple pregnancy, cardiovascular diseases, diabetes, renal diseases, thyroid disorders, pulmonary diseases, autoimmune disorders, Pre-eclampsia, placental abruption, premature rupture of membranes, hepatitis, AIDS and other problems; not using special drugs which affect birth weight during pregnancy.

Willing to participate in the research

### 3.2. Infants

**Figure 1.**
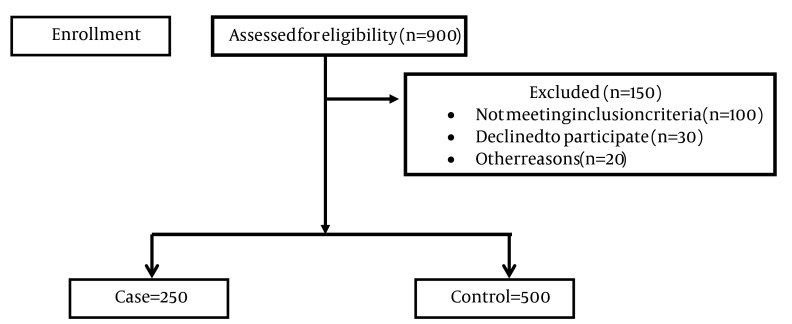
Consort Flow Diagram

Birth weight of more or less than 2500 g with no known medical problems like congenital abnormalities, cardiac or pulmonary diseases and so on. After obtaining permission from university and hospital authorities, we presented the required information to the study population and convinced them to cooperate. Then the questionnaire was filled out by a team of trained people. The questionnaire was filled out in the following way: first, the questioner or researcher selected the mothers with inclusion criteria in the delivery room and monitored them until delivery. At the time of delivery, the researcher or questioner went to the delivery room, and immediately after delivery, if the infant had no medical problems like congenital disorders, cardiac-pulmonary diseases, etc., and its weight was below 2500 g using the scale in the delivery room, it was placed in the case group. If it weighed between 2500 and 4500 g, it was placed in the control group (Figure 1). 

Measurement accuracy of all scales in delivery rooms was measured by the researcher as follows: in order to determine scale reliability, a standard weight (control weight of 100 g) was used to control and calibrate the scale after every 10 samples. After transferring the mother to the general care unit and in case she was in good conditions and willing to participate in the study, she was asked to fill out a consent letter. Then, the part of questions related to the patient’s file including laboratory test results, ultrasound examination and so on was completed by the researcher by checking mother’s medical file. Another part which included demographic questions and those related to lifestyle were filled out by interviewing the mother. In this part, information of other important factors which affect low weight including socio-economic status (family's income, education, occupation and number of family members, husband’s occupation), pregnancy variables (age, number of pregnancy) and mother’s health status (blood pressure, weight gain and BMI) was gathered and recorded in the results.

Based on MET scale, physical activity may be defined in terms of time, amount and intensity. Hence, to calculate time duration of physical activities of mothers during pregnancy, the time spent on each activity (according to mothers report) was calculated in terms of hours per week and then, For measuring intensity of activities according to MET scale, MET value of each activity (based on MET table)(22) was multiplied by time period of doing that physical activity in hour per week and thus intensity of each physical activity was obtained. Then, the intensity of physical activities was summed to obtain the amount of activities. Those activities with MET of below 1.5 were called sedentary, between 1.5 and 3 were light, within 3-6 were average and or more than 6 were severe in terms of intensity(23, 24). In order to respect ethical considerations, the study was conducted upon receiving the consent of chancellors of University of Tehran, Shahid Beheshti University and General Department of Social Security for their affiliated hospitals. Moreover, prior to the study, pregnant women signed an informed consent form after they were informed of the objectives of the study and were assured that their information would remain confidential and they could withdraw from the study whenever they liked. The study was approved by Welfare and Rehabilitation Sciences University and research center for social determinant of Health Ethics Committee. The data were analyzed and interpreted in SPSS software (version 16) using t-test, Chi-square and logistics regression in confidence interval of 95% and P < 0.5.

## 4. Results

In this study, the two groups (500 control pregnant women with 2500-4500 g infants and 250 case women with infants weighing less than 2500 g) had no significant difference in terms of mean of age, BMI, pregnancy age, pregnancy intervals and family income. But, mothers’ mean of weight increase during pregnancy was significantly different in the two groups (P = 0.002). Mean of birth weight in two groups, case and control, respectively were 2.183 ± 4.24 and 3.243 ± 3.76 and between them according sex weren’t significantly different (Table 1). 

**Table 1.** Comparing Some Neonatal Information in the Two Groups of Normal Weight and Low Weight Infants 2012

Variables	Normal	LBW	P value
**BirthWeight, Mean ± SD**	3.243 ± 3.76	2.183 ± 4.24	
**Gender, No (%)**			0.098 ^a^
Male	267 (53.4)	117 (46.8)	
Female	233 (46.6)	133 (53.2)	

^a^ X^2^

In evaluating educational level, chance of delivering low birth weight infant in illiterate mothers was three times as much as that in educated mothers (P = 0.031, OR = 3.27). Husbands’ occupation and mothers’ employment were among other factors which were related to infants' low birth weight in that if the husband was unemployed, chance of this outcome was 4.5 times higher (P < 0.001, OR = 4.49). Also, mothers' employment increased chance of delivering infants with low birth weight by 5.4 times (P < 0.001, OR = 5.4). Table 2, 3 shows participants’ individual and social characteristics in both groups. 

**Table 2.** Comparing Some Personal-Social Factors of Research Units in the Two Groups of Normal Weight and Low Weight Infants 2012

Variables	Normal ^a^, Mean ± SD	LBW ^b^, Mean ± SD	t test
**Age, yr, **	27.34 ± 5.2	27.95 ± 5.3	P = 0.130
**Weight Before Pregnancy, kg**	63.07 ± 11.65	63.94 ± 11.47	P = 0.331
**Weight Gain, kg ^c^**	13.92 ± 5.29	12.68 ± 5.06	P = 0.002
**BMI, kg, m^2^**	24.25 ± 4.14	25.54 ± 4.08	P = 0.350
**HB**	11.98 ± 1.1	11.97 ± 1.9	P = 0.941
**HCT**	36.35 ± 3.6	4.1 ± 36.98	P = 0.221
**Interval of Pregnancy (mount)**	5.47 ± 1.17	1.39 ± 5.22	P = 0.060
**Income, RLS**	6190000 ± 250.19	6320000 ± 34.271	P = 0.501
**Residential Density per Unit**	26.9 ± 12.65	28.03 ± 12.99	P = 0.250

^a^ Normal weight: infants weighing 2500 g and more

^b^ Low weight: infants weighing less than 2500 g

^c^ significant

**Table 3.** Comparing Some Social Factors ****Of Research Units in the Two Groups of Normal Weight and Low Weight Infants 2012

Variables	Normal ^a^, No (%)	LBW ^b^, No (%)	t test, X^2^
**Educational ^c^**			P = 0.031, OR = 3.273, CI = 1.05-10.11
Illiterate	5 (1)	8 (3.2)	
Literate	495 (99)	242 (5.8)	
**Husbands' Job ^c^**			P < 0.001, OR = 4.49, CI = 2.15-9.37
Unemployed	12 (2.2)	23 (9.2)	
Employed	488 (97.8)	227 (90.8)	
**Mothers Job ^c^**			P < 0.001, OR = 5.35, CI = 3.34-8.58
Employed	29 (5.8)	62 (24.8)	
Housekeeper	471 (94.2)	188 (75.2)	

^a^ Normal weight: infants weighing 2500 g and more

^b^ Low weight- infants weighing less than 2500 g

^c^significant

In this investigation, in terms of time spent on physical activity (week/hour), the mean time spent on doing physical activity at home and leisure was significantly different, in that in the control group, the time spent on physical activity at home was less than that of the case group (18.97 vs. 22.42), but time of doing leisure physical activity was more in the control group than in the case group (8.06 vs. 6.32) (P = 0.038 and 0.003). These results showed a direct relationship between the time spent on physical activity at home and an inverse relationship between the times spent on leisure physical activity on the one hand and low birth weight on the other. There was no significant relationship between mean time of physical activity along with total physical activity and birth weight in the two groups. In terms of mean level of their physical activity based on MET, no significant difference was observed between the amount of physical activity in the two groups, but there was a significant difference between physical activity at home, leisure physical activity and total amount of their physical activity (Table 4). 

**Table 4.** Distribution of Research Units in Two Groups of Normal and Low Weight Infants Based on Mean and Standard Deviation of the Amount of Physical Activities in Terms of MET 2012

Neonate weight Activity, MET	Normal, Mean ± SD	LBW, Mean ± SD	t test
**Exercise/Sport**	13.10 ± 17.03	14.48 ± 14.38	P = 0.271
**Home Activities**	39.36 ± 32.39	46.45 ± 27.01	P = 0.003
**Leisure Time Activities**	10.49 ± 15.54	8.22 ± 13.22	P = 0.038
**Total Activities**	60.52 ± 43.03	66.93 ± 38.81	P = 0.040

As far as intensity was concerned, mean time of activity using 3-6 MET scale did not show any difference between the two groups; however, mean time of physical activity with light intensity (P = 0.002) in the case group and sedentary (P = 0.040) in the control group was significantly higher (Table 5). 

**Table 5.** Distribution of Research Units in Two Groups of Normal and Low Weight Infants Based on Mean and Standard Deviation of the Intensity of Physical Activities in Terms Of MET 2012

Neonate Weight Intensity	Normal, Mean± SD	LBW, Mean± SD	t test
**Moderate Activity**	3.27 ± 4.25	3.62 ± 3.59	P = 0.310
**Light Activity**	9.84 ± 8.13	11.61 ± 6.75	P = 0.002
**Sedentary Activity**	3.49 ± 5.18	2.74 ± 4.40	P = 0.040

Variables which affected low birth weight based on independent t-test were entered into the multiple regression model. The results of multiple regression model in terms of the effect of above variables on low birth weight are presented in Table 5. The results demonstrated that among individual social variables, each kilogram of mother's weight gained during pregnancy decreased the chance of low birth weight by 0.96 (P = 0.007); in contrast, variables like blood pressure (OR = 2.42, P < 0.001), husbands' unemployment (OR = 4.11, P < 0.001) and lack of husbands’ help for at home (OR = 1.46, P = 0.030) was related with increased chance of low birth weight. Moreover, increased family size and time of physical activity (exercise, leisure and home activity) increased the chance of low birth weight (P = 0.030 and P =0.0004, respectively). Based on the results of regression test, each hour of increased physical activity and home activity per week increased chance of low birth weight by 2.20 and 1.44 times, respectively, but one hour increase of leisure activity decreased the probability of delivering low birth weight infants by 0.32 (Table 6). 

**Table 6.** The Relationship Between Birth Weight Physical Activity Domain and Individual Social Characteristics of Research Units Based on the Infants' Weight 2012

Variables	Normal, No (%)	LBW, No (%)	B	OR (95% CI)
**BP, mm, hg**				
Normal	453 (90.6)	195 (78)		1
14.9 ≥	47 (9.4)	55 (22)	0.88	2.43 (1.55 - 3.78)
**Parity**				
0	230 (46)	129 (51.6)		1
2 - 3	245 (49)	97 (38.8)	0.32	0.72 (0.34 - 1.50)
4 ≤	25 (5)	24 (9.6)	0.10	1.10 (0.44 - 2.77)
**Family Size**				
2	245 (49)	131 (52.4)		1
3	174 (34.8)	58 (23.2)	0.39	1.48 (0.91 - 2.43)
4 ≥	81 (16.2)	61 (24.4)	0.53	1.72 (0.95 - 0.36)
**Education**				
Literate	495 (99)	242 (96.8)	1.14	1
Illiterate	5 (1)	8 (3.2)		0.32 (0.09 - 1.1)
**Employment Status Of Husband**				
Employed	488 (97.8)	227 (90.8)		1
Unemployed	12 (2.2)	23 (9.2)	1.4	4.11 (0.11 - 0.55)
**Husbands’ Helping Around the House**				
Yes	296 (59.2)	114 (45.6)		1
No	204 (40.8)	32 (52.8)	0.38	1.46 (0.48 - 0.97)
**Weight gain, kg, Mean ± SD**	13.92 ± 5.29	12.68 ± 5.06	0.045	0.96 (0.92 - 0.98)
**Physical Activity, h, w, Mean ± SD**				
Exercices	2.9 ± 3.76	3.30 ± 3.25	0.79	2.20 (1.28 - 3.8)
Home Activities	18.97 ± 15.64	22.42 ± 13.09	0.36	1.44 (1.12 - 1.84)
Leisure Time	8.06 ± 11.95	6.32 ± 10.17	0.27	0.32 (1.07 - 1.62)

## 5. Discussion

The present study showed that mean time spent on exercise and home physical activities was in direct relationship with chance of low birth weight so that increasing the time spent on the mentioned activities led to increased chance of delivering a low birth weight infant. In developing countries, women are responsible for house chores and babysitting in addition to outdoor work and are thus exposed to undesirable pregnancy complications such as low birth weight(25). The important point is that doing the mentioned activities by women has not been considered an effective determinant in their lifestyle. Only the activities like sports have been considered influential and protective factors; however, lifestyle and its dimensions include all aspects of people’s lives. Mothers who take care of children at home and are responsible for housework might not go to the gym. Moreover, they consume the same amount of energy or even more according to MET scale, and face other factors like stress and worry. Launer et al. found that mothers who do housework alone had 1.7 times more chance of delivering low birth weight infants. In their study, Takto et al. found a negative relationship between the time spent on physical activity at home and low birth weight(2), but Jahromi et al. found no relationship between physical activity at home and low birth weight; they instead found a positive relationship between doing physical activity during pregnancy and low birth weight, which contradicted the findings of the present study. In this study, a negative relationship was observed between physical activity during pregnancy and low birth weight. In the study by Jahromi et al., 97% of mothers used to do sport activities before pregnancy and continued them during pregnancy, which is one of the most important reasons for the difference in the results of these two studies(26). Researchers believe that doing physical activity is probably related to reduced uteroplacental blood flow, increased body temperature, decreased available blood glucose and increased catecholamine secretion which can lead to increased uterine contraction. The highest concern of doing physical activities during pregnancy is the decreased oxygen, glucose and other required materials for fetus(26). There is also the hypothesis that doing physical activities during pregnancy independently affects mothers’ weight gain during pregnancy and consequently infants' birth weight. In a systematic study, some researchers have also confirmed this hypothesis, but some others have reported no relationship(19). Accordingly, in logistic regression test, no difference was observed in the results of physical activities and home activities on birth weight although all the factors affecting birth weight like weight gain during pregnancy were considered.

In this study, leisure physical activity was found to have a protective effect on birth weight. This finding concurs with the results of many studies such as Domingues and Barros and of Takito et al., who referred to a positive protective relationship between leisure physical activity and birth weight(2). It seems that leisure physical activities like listening to music, studying, resting, etc. reduce mothers’ stress, increase their weight gain, and increase blood supply to fetus, so they affect infants' birth weight. Probably, one of the most important effects of listening to music, studying or attending religious rites would be decreased stress and its unfavorable effects in pregnancy. Kafali et al. showed that mothers’ listening to music increased movement of the fetus and its heartbeat rate(27). Tabarro also found that the women who listened to music in their delivery room suffered less pain and stress and had a better delivery experience (28). Attending religious and spiritual ceremonies also managed pregnancy complications via decreasing stress and worry and establishing self-confidence (29).

In this study the correlation between BP and low birth weight was significant. High Blood pressure can Be dangerous for both Mather and the fétus. Women with pre-existing, or chronic, high blood pressure are more likely to have certain complications during pregnancy than those with normal blood pressure. However, some women develop High Blood pressure while they are pregnant and due some adverse outcome such as low birth weight, preterm birth, Intrauterine Growth restriction (IUGR), Intrauterine Fetal Death (IUFD). This finding concurs with the results of some studies (12, 29). Various studies have addressed the relationship of education and income level of families with their physical activities, none of which have sufficiently studied the relationship between socio-economic factors and physical activity or have ignored some of its dimensions(30). Thus, considering many challenges with regard to socio-economic inequalities and their effects on pregnancy outcomes, this study tried to address the effects of some individual social characteristics of research units on the chance of low birth weight. As mentioned in the findings section, only the individual social variables which were significant in the tests were entered into the logistic regression analysis. Among them, the only variables that had no significant difference in this analysis were education and number of mothers’ pregnancies. These findings were in line with those of Ramezanzadeh et al. who found no relationship between mothers’ education and pregnancy outcomes(31). Probably, one of the most important reasons that mothers’ education had no effect on this outcome in the present study and other mentioned works was that education level of all participating mothers was identical. However, some researchers believe that mothers’ education either directly or indirectly affects birth weight through improving health status, self-care and accessing financial resources(32).

In logistic regression test, occupation was found to have a négatives effect on birth weight. This finding concurs with the results of the study of Needhammer et al. (2009) it was found that more than 40 hours of work per week and shift work increase risks of low birth weight incidence, small for gestational age (SGA), and preterm labor. They also found that part-time work can be a preventative factor for preterm labor (33). Another economic factor affecting birth weight in this study was the number of family members (those who lived in the same house with the family) so that the more the people living in the family, the greater the chance of delivering a low weight infant. This finding was in line with that of Qawami et al. (34), which could be due to lack of enough rest, more activity and stress, etc. In some studies, a relationship has been found between various psychological problems and large families; in these families, there was less tendency for pregnancy, caretaking and pregnancy support, all of which could have a role in emergence of adverse pregnancy complications (35). An important positive point in this study was matching the study groups based on confounding variables like gestational age, family's income level, area of place of residence and body mass index.

In this study, mothers were interviewed shortly after delivery; thus, there is the possibility of their frustration after delivery or pregnancy result might have an influence on their responses. Also, the researches only studied the variables that had the possibility of investigation via interviewing with mothers. There might have been other elements as well which affect birth weight but have not been taken into consideration in this study. So, other studies should be done in this field considering the mentioned conditions. The findings showed that increased athletic physical activities and home activities are directly related to low birth weight, even after considering other effective confounding factors. Therefore, holding preventive educational programs like consulting classes for mothers and their spouses for teaching appropriate physical activities in this period and also spouses’ cooperation in house chores could be very helpful.
